# Draft genome assemblies of one *Lactobacillus gasseri* strain and two *Lactobacillus jensenii* strains isolated from voided urine samples

**DOI:** 10.1128/mra.00615-24

**Published:** 2024-07-22

**Authors:** Alex Kula, Natalie Stegman, Catherine Putonti

**Affiliations:** 1 Department of Biology, Loyola University Chicago, Chicago, Illinois, USA; 2 Bioinformatics Program, Loyola University Chicago, Chicago, Illinois, USA; 3 Department of Microbiology and Immunology, Loyola University Chicago, Maywood, Illinois, USA; Department of Biological Sciences, Wellesley College, Wellesley, Massachusetts, USA

**Keywords:** *Lactobacillus*, *Lactobacillus gasseri*, *Lactobacillus jensenii*, female urobiome, urinary tract

## Abstract

We present the draft genome for three *Lactobacillus* strains isolated from female urine specimens: *Lactobacillus gasseri* UMB1673, *Lactobacillus jensenii* UMB1855, and *Lactobacillus jensenii* UMB5069. Focusing on strains within the female urinary microbiome can provide a more well-rounded understanding of the microbial community and its influence on health and disease.

## ANNOUNCEMENT

The urobiome is a complex and dynamic community of microorganisms that inhabit the urinary tract (1, 2). *Lactobacillus* species are well-known inhabitants of the gut and vagina, where they contribute to a healthy balance of microorganisms (3). Notably, *Lactobacillus gasseri* and *Lactobacillus jensenii* are part of the flora of the urobiome in healthy females, and they compete with harmful bacteria for nutrients and space, thus limiting their ability to establish (4). *L. gasseri* and *L. jensenii* are known for their beneficial properties, including their ability to produce lactic acid and antimicrobial compounds in the urinary tract and inhibit the growth of pathogenic bacteria (5, 6). The findings for these three *Lactobacillus* strains, which were isolated from urine samples from separate female participants, highlight the prevalence of this genus in the female urobiome.

All three of the strains were collected as part of an IRB-approved study (LU204197) of females with type 2 diabetes (7); voided urine was obtained from participants at Loyola University Medical Center (Maywood, IL). Using the enhanced quantitative urine culture method, strains were isolated and stored at −80°C (6). We obtained these strains from the Loyola Urinary Education and Research Collaborative collection. Each strain was streaked onto de Man-Rogosa-Sharpe (MRS) agar plates (8) and incubated with 5% CO_2_ at 35°C for 48 hours. A single colony was selected and grown in MRS medium in 5% CO_2_ at 35°C for 48 hours. DNA was extracted from liquid culture using the Qiagen DNeasy Blood and Tissue Microbial Kit following the manufacturer’s protocol for Gram-positive bacteria. DNA was sent to SeqCoast Genomics LLC (Portsmouth, NH, USA), where library preparation and sequencing were performed. DNA was prepared for whole-genome sequencing using an Illumina DNA Prep Tagmentation Kit and unique dual indexes. Sequencing was performed on the Illumina NextSeq2000 platform using a 300-cycle Flow Cell Kit, producing 2 × 150 bp paired reads. Read demultiplexing, read trimming, and run analytics were performed using DRAGEN v3.10.12. The website BV-BRC v3.36.16.5 (9) was utilized to assemble all strains. The assembly “auto” option was selected, and BV-BRC alignment service performed the following steps. Raw reads were trimmed using Trim Galore v0.6.5dev (https://github.com/FelixKrueger/TrimGalore) and normalized using BBNorm (part of BBMap, https://jgi.doe.gov/data-and-tools/software-tools/bbtools/; v 19 October 2017). The processed reads were assembled with Unicycler v0.4.8 (10) and then polished via Pilon v1.23 (11); two rounds of Pilon were performed. Finally, the quality of assemblies was determined by BV-BRC using QUAST v5.2.0 (12). The draft genome assemblies were annotated using the NCBI Prokaryotic Genome Annotation Pipeline v6.7 upon submission (13). Unless otherwise stated, default parameters were used.

Table 1 displays the sequencing and genome statistics for all three strains. Further research on these strains’ properties could provide valuable insights into the strain’s contributions to urinary health.

**TABLE 1:** Genome sequencing assembly statistics

*S*train	No. of raw reads	No. ofcontigs	Contig N50 (bp)	GC content (%)	Coverage (×)	Assembly length (bp)	SRA accession no.	WGS project no.
*L. gasseri* UMB1673	1,325,020	17	576,579	35.6	218.10	1,787,187	SRR28574801	JBBUVK01
*L. jensenii* UMB1855	1,326,173	46	87,588	34.4	209.30	1,859,671	SRR28574802	JBBVUL01
*L. jensenii* UMB5069	150,564	46	65,048	34.6	255.66	1,726,099	SRR28574803	JBBVUM01

## Data Availability

Table 1 lists the accession numbers for the whole-genome sequencing project and the raw sequencing reads for the three strains presented here.
